# Accelerated CXL Versus Accelerated Contact-Lens Assisted CXL Treatment for Progressive Keratoconus—A 3-Year Retrospective Comparative Follow-Up

**DOI:** 10.3390/jcm14207141

**Published:** 2025-10-10

**Authors:** Anna Bunin, Shmuel Kagasov, Nir Amitai, Tomer Kerman, Ran Matlov Kormas, Soosan Jacob, Raimo Tuuminen, Liron Kravitz, Boris Knyazer

**Affiliations:** 1 Department of Ophthalmology, Soroka University Medical Center and Faculty of Health Sciences, Ben-Gurion University of the Negev, Beer-Sheva 84101, Israel; lironk8@gmail.com (L.K.); bknyazer@gmail.com (B.K.); 2 Faculty of Health Sciences, Ben-Gurion University of the Negev, Beer-Sheva 8410501, Israel; amitaini@post.bgu.ac.il (N.A.); kerman@post.bgu.ac.il (T.K.); raimo.tuuminen@helsinki.fi (R.T.); 3 Clinical Research Center, Soroka University Medical Center, Beer-Sheva 84101, Israel; 4 Department of Ophthalmology, Kaplan Medical Center, Rehovot 76100, Israel; ran_kormas@yahoo.com; 5 Dr. Agarwal’s Refractive and Cornea Foundation, Dr. Agarwal’s Eye Hospital, Chennai 600018, India; dr_soosanj@hotmail.com; 6 Helsinki Retina Research Group, Faculty of Medicine, University of Helsinki, 00014 Helsinki, Finland; 7 Eye Centre, Kymenlaakso Central Hospital, 48210 Kotka, Finland

**Keywords:** cross-linking, keratoconus, thin corneas

## Abstract

**Background:** Contact lens-assisted corneal cross-linking can be used to treat keratoconus in patients with thin corneas under measuring less than 400 µm. This study compares the long-term clinical and tomographic outcomes between accelerated corneal cross-linking (A-CXL) and accelerated contact lens-assisted corneal cross-linking (A-CACXL). **Methods:** Patients who underwent either A-CXL or A-CACXL protocol due to progressive keratoconus were enrolled in this retrospective cohort study conducted between January 2015 and December 2018. The control group (patients with minimum corneal thickness of at least 400 µm, comprising 32 eyes of from 32 patients) was treated with A-CXL, whereas the treatment group (patients that had minimum corneal thickness after epithelial removal below 400 µm; 30 eyes of from 30 patients) underwent the A-CACXL protocol. Clinical and tomographic data were obtained from a 3-year follow-up period. **Results:** At 3 years, both groups represented a significant gain in best-corrected visual acuity (from 0.32 to 0.18 LogMAR units for A-CXL, *p* = 0.001; from 0.51 to 0.33 LogMAR units for A-CACXL, *p* = 0.037). Furthermore, postoperative tomographic parameters (Kmax, Kmean, or corneal astigmatism) were comparable between the two protocols. Progression of keratoconus was halted among 87% of eyes in the A-CXL group and among 73% of eyes in the A-CACXL group (*p* = 0.2). **Conclusions:** A-CACXL treatment is an effective and safe option for patients with keratoconus and thin corneas, yielding long term outcomes comparable to those of A-CXL treatment for patients with a minimum corneal thickness of 400 µm following a 3-year follow-up.

## 1. Introduction

Keratoconus (KC) is an ectatic disorder characterized by bilateral, yet often asymmetric progressive thinning and steepening of the cornea, resulting in irregular astigmatism and subsequent loss of visual acuity [1]. Available options for managing KC are considered based on the stage of the disease and the extent of its progression. In case of a stable disease, we prioritize vision correction. However, if the disease progresses, the focus is on halting its progression [2]. Corneal collagen cross-linking (CXL), originally described by Wollensak et al. [3] is a minimally invasive outpatient treatment shown to effectively halt the advancement of KC [4]. By inducing additional covalent bonds within the stromal collagen, CXL increases biomechanical rigidity of the cornea, a mechanism supported by ex vivo studies using tensile and enzymatic digestion assays [5].

Recent studies have indicated that accelerated CXL (A-CXL) using 9 mW/cm^2^ for a 10 min protocol may halt keratoconus progression similarly to standard CXL (S-CXL) [6,7]. A major limitation of CXL in thin corneas lies in the risk of endothelial damage from ultraviolet-A (UVA) irradiation due to the corneal stroma being insufficiently thick to absorb and attenuate the light. Wollensak et al. reported that a combination of riboflavin with UVA has significantly caused more damage than UVA irradiation alone [8]. Moreover, the cytotoxic threshold for riboflavin-sensitized UVA exposure is exceeded when the de-epithelialized corneal thickness is below 400 μm [8].

Consequently, standard treatment is generally restricted to patients with corneas measuring at least 400 μm after epithelial removal. In our previous work, we observed that in southern Israel, approximately 8% of adult KC patients aged 18 and older, presented with a minimum corneal thickness below 400 μm and 39% below 450 μm [9]. The high incidence of thin corneas among KC eyes led to the development of alternative CXL protocols and methods for treating such eyes. Hypo-osmolar riboflavin was introduced by Hafezi et al. in 2009, inducing stromal swelling, and leading to an increase in corneal thickness [10]. Alternative treatment strategies include iontophoresis [11], stromal lenticules [12], epi-on (transepithelial) CXL [13], nomograms for treatment of KC patients with thin cornea [14], and contact lens-assisted CXL (CA-CXL) [15]. The CA-CXL technique, first described by Jacob et al. in 2014, utilizes a riboflavin-soaked contact lens to artificially thicken the cornea from 96 to 124 µm, thereby eliminating the need for corneal swelling [15]. Mazzotta et al. subsequently showed that this approach generates a well-defined deep stromal demarcation line without evidence of endothelial toxicity [16]. In our previous study, accelerated contact lens-assisted corneal cross-linking halted keratoconus progression and achieved regression after a 1-year follow-up, in a similar fashion to accelerated corneal cross-linking [17]. In this study, we extended the follow-up period of our previously published paper in order to provide a longer-term comparison of treatment outcomes at 3 years [17]. According to current knowledge, our study is the first to examine and compare the clinical and tomographic characteristics of patients with keratoconus and MCT below 400 µm with those having MCT of 400 µm or greater following an A-CXL protocol over a 3-year follow-up.

## 2. Materials and Methods

This single-center retrospective cohort study of consecutive patients with progressive keratoconus who underwent either accelerated contact lens-assisted CXL (A-CACXL) or accelerated corneal cross-linking (A-CXL) at the Department of Ophthalmology, Soroka University Medical Center (Beer-Sheva, Israel) between January 2015 and December 2018. Only patients with at least three years of postoperative follow-up were included in the study. Institutional Review Board approval was obtained from Ben-Gurion University of the Negev (SOR-0322-24) in 14 January 2025, and all procedures adhered to the principles outlined in the Declaration of Helsinki.

### 2.1. Study Participants

#### Eligibility Criteria

Progression of keratoconus was defined as any of the following: ≥1.5 diopters (D) increase in mean keratometry, ≥1.0 D increase in maximum keratometry (Kmax), or ≥5% reduction in central corneal thickness, confirmed on repeat examination with Scheimpflug tomography (Pentacam HR, OCULUS, Wetzlar, Germany) after at least 12 months follow-up [15,18,19,20].

Patients were eligible for inclusion if they were 18 years of age or older, had documented keratoconus progression (as defined by the criteria above and confirmed on repeat tomography), and had undergone either accelerated corneal cross-linking (A-CXL) or accelerated contact lens–assisted CXL (A-CACXL). Additionally, only individuals who had completed a minimum of three years of postoperative follow-up and provided signed informed consent prior to the procedure were included in the study. To avoid inter-eye correlation bias, we included only one eye from each eligible patient [21].

We excluded patients from our research in case of previous ocular surgery in the examined eye, a history of corneal disease other than keratoconus, corneal curvature above 75 diopters, pregnancy or breastfeeding, a history of autoimmune or systemic diseases (such as, rheumatoid diseases, diabetes), presence of corneal scar, riboflavin sensitivity or sensitivity to other substance applied during CXL procedure. Patients with a minimal corneal thickness (MCT) less than 400 µm who underwent bandage contact lens application prior to surgery did not undergo this procedure. Moreover, we excluded patients with a follow-up time shorter than 3 years.

The patients were divided into two groups: the A-CACXL group, with a MCT of less than 400 µm, and the A-CXL group, with a MCT of 400 µm or greater. A statistical comparison was applied only to patients with data available for at least 3 years following their CXL procedure.

### 2.2. Data Collection

Medical records were reviewed for demographic and clinical data, including: age, sex, ethnicity, date of procedure, uncorrected distance visual acuity (UDVA), best-corrected visual acuity (BCVA), K-max, anterior and posterior keratometric indices, MCT, index of surface variance (ISV), index of height decentration (IHD), and Belin–Ambrosio enhanced ectasia deviation (BAD-D). All keratometric and tomographic parameters were measured using Pentacam HR; Oculus Optikgeräte. Data extraction was independently performed by two investigators (AB and SK), with adjudication by a third (BK) when necessary.

### 2.3. Statistical Analysis

Continuous variables were described using means, medians, standard deviations (SDs), and ranges, while categorical variables were presented as frequencies and percentages. Univariate analysis was initially conducted to identify differences in baseline characteristics between the groups. For continuous variables, *t*-tests were used when the data was normally distributed, while the Mann–Whitney U test was performed for non-normally distributed data. Chi-Square or Fisher’s Exact tests were employed for categorical variables. Additionally, separate paired longitudinal analyses were performed for each group to compare baseline with follow-ups at 12 months and 3 years, assessing the progression of parameters over time. Depending on their distribution, continuous variables were analyzed using paired *t*-tests or paired Wilcoxon tests. Kmax progression was defined as an increase in Kmax by at least 1 diopter, and Kmax regression as a decrease by at least 1 diopter. All statistical tests were two-tailed, with significance set at a *p*-value of less than 0.05. Analyses were performed using R software, version 4.3.1.

### 2.4. Surgical Technique

Table 1 outlines the characteristics of the A-CACXL surgical approach according to established protocol nomenclature [18]. All procedures were performed by the same surgeon (B.K) and under sterile conditions in an operating room using topical anesthesia with 0.4% benoxinate hydrochloride drops. MCT was initially confirmed using an ultrasound pachymeter (PachPen; Accutome, Malvern, PA, USA) with three repeated measurements at the thinnest corneal location as identified by tomography. Following the mechanical removal of the central 8 mm of the corneal epithelium, the MCT was re-evaluated. If the post-debridement thickness was less than 400 µm, the eye was treated using the A-CACXL protocol. This protocol entailed the administration of an iso-osmolar 0.1% riboflavin solution (Medio-Cross 0.1%; Peschke Meditrade, Hunenberg, Switzerland) at two-minute intervals for a total duration of 30 min, while simultaneously soaking a 90-µm-thick daily disposable, UV-filter–free soft contact lens (SofLens; Bausch & Lomb, Rochester, NY, USA) in the same riboflavin solution. After confirming adequate stromal saturation by the appearance of flare in the anterior chamber, the contact lens was applied on the cornea, and MCT was reverified to ensure it exceeded 400 µm. The A-CACXL procedure was conducted utilizing a methodology akin to that outlined by Jacob et al., with the main distinction being the accelerated nature of the process [15].

UVA irradiation was applied at 365 nm with an intensity of 9 mW/cm^2^ for 10 min, delivering a total fluence of 5.4 J/cm^2^, using a LightLink-CXL device (LightMed, San Clemente, CA, USA). Riboflavin drops were applied every 5 min during irradiation. Patients were instructed to fixate directly on the light source while the surgeon ensured centration. For eyes with an MCT of 400 µm or greater, the A-CXL protocol was performed in the same manner, except that the contact lens was excluded during irradiation. Following the procedure, all eyes received a bandage contact lens to support epithelial healing.

### 2.5. Postoperative Follow-Up

Patients were prescribed topical ofloxacin 0.3% four times daily for 10–12 days and topical dexamethasone 0.1% tapered over a period of one month. Preservative-free lubricants were recommended as needed. Scheduled follow-ups occurred on day 1, week 1, and at 1 month, 6 months, 1 year, and 3 years. The bandage lens was removed after epithelial healing, typically at the week 1 visit.

### 2.6. Main Outcome Measures

The main outcome measures were to investigate the A-CACXL efficacy by comparing the UDVA, BCVA, and Kmax values between the groups at the last visit after 3 years. The secondary outcome measures were tomographic parameters at the last visit, progression, and regression assessment.

## 3. Results

Overall, our study included 32 eyes of 32 patients with a MCT of 400 µm or greater and 30 eyes of 30 patients with a MCT of less than 400 µm. The mean age was 21.8 ± 4.5 years in the control group and 25.2 ± 7.1 years in the A-CACXL (*p* = 0.022). In the A-CXL group 57% (n = 17) were males and 43% (n = 13) were females. The clinical and demographic characteristics of participants are described in Table 2. Most visual and keratometric characteristics of the A-CACXL group demonstrated more advanced stages of keratoconus than the A-CXL group.

### 3.1. Efficacy

For UDVA, the control group showed significant improvement from baseline to the 3-year follow-up visit (0.63 ± 0.41 to 0.44 ± 0.28 LogMAR units; *p* = 0.031, Table 3). The case group exhibited a trend of improvement, although this was insignificant at the 3-year follow-up visit (0.93 ± 0.58 to 0.66 ± 0.51 LogMAR units; *p* = 0.056, Table 3). A significant improvement in BCVA was observed both for the control group at the 3-year follow-up visit (0.32 ± 0.18 to 0.18 ± 0.15 LogMAR units; *p* = 0.001, Table 3) and the case group (0.51 ± 0.30 to 0.33 ± 0.34 LogMAR units; *p* = 0.037, Table 3).

Keratometric measurements revealed that the A-CXL-treated control group exhibited a stable Kmax, with values decreasing slightly from 55.1 ± 4.2 D to 54.5 ± 3.9 D (*p* = 0.5, Table 3). Alike, the A-CACXL treated case group showed only minimal changes in Kmax values from 61.0 ± 6.0 D to 60.2 ± 6.6 D (*p* = 0.8, Table 3). Similarly, the Kmean front values remained stable in both groups.

The BAD-D parameter remained stable, with no significant changes in either group (Table 3). ISV showed an improvement for both groups within the 3-year follow-up. Control group improved from 90 ± 29 to 84 ± 28 µm; *p* = 0.4, whereas the case group improved from 108 ± 31 to 102 ± 34 µm; *p* = 0.5. The IHD of the control group changed from 0.11 ± 0.05 to 0.15 ± 0.13 µm (*p* = 0.1), while in the case group, it changed from 0.12 ± 0.06 µm to 0.13 ± 0.08 µm (*p* = 0.6, Table 3).

### 3.2. Regression and Progression

**Figure 1:**
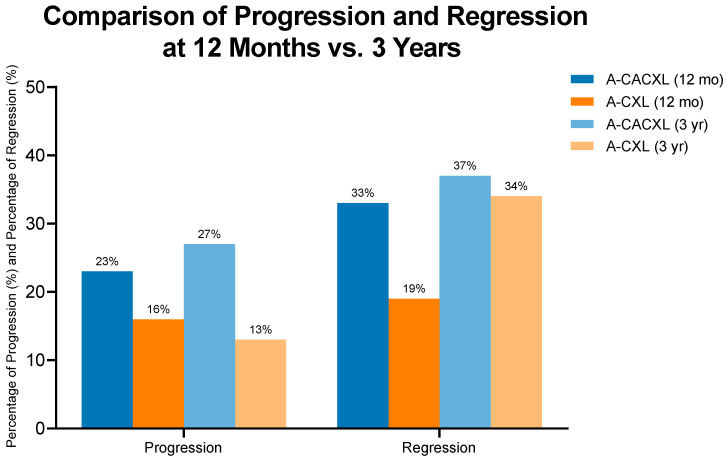
Progression and regression of 1-year and 3-year follow-up in study populations.

Regression was defined as a decrease of at least 1.00 D in the Kmax value; progression was defined as an increase of at least 1.00 D in the Kmax value. As shown in Figure 1, progression was observed in 23% of the treatment group at the 12-month visit, compared to 16% in the control group. Regression rates at 3 years were 37% (11 out of 30) for the treatment group and 34% (11 out of 32) for the control group (Figure 1, Table 4). Progression rates at 3 years were 13% (4 out of 32) for the control group and 27% (8 out of 30) for the case group (*p* = 0.2, Figure 1, Table 4).

### 3.3. Safety

During the follow-up period, no instances of infectious keratitis or delayed epithelial healing were observed. Corneal haze developed in two cases and resolved completely with topical dexamethasone 0.1%. In both groups, no deterioration in UDVA or BCVA was noted compared with preoperative values. No signs of endothelial compromise, such as corneal edema or Descemet’s membrane folds, were observed throughout the follow-up. Endothelial cell density measurements were not performed at the three-year time point.

## 4. Discussion

Keratoconus patients exhibit a reduced thickness of the stromal layer, which leads to several limitations when performing either a standard or accelerated CXL procedure. A corneal thickness of at least 400 µm is required to perform CXL safely by using the Dresden protocol [19]. Performing CXL procedure on thin corneas increases the risk of damaging the endothelial layer and other ocular structures, such as the lens [20]. Therefore, thin corneas often need modified protocols to achieve effective and safe results.

CACXL is a modified CXL procedure that forms additional pre-corneal layers using riboflavin film and a contact lens, achieving a thickness of at least 400 µm for irradiance attenuation. The benefit of this technique lies in its independence from corneal swelling properties, the simplicity of the procedure, and its safety [14,21].

The biochemical efficacy of CACXL and standard CXL was examined by Wollensak et al. and Zhang et al., who compared both procedures on enucleated porcine eyes. CACXL showed 70% of the stiffening achieved by the standard CXL group [22,23]. On the other side, Kling et al. found that CXL has a more effective biomechanical influence on thin murine corneas than on thicker porcine corneas, due to a greater oxygen bioavailability in thinner corneas [24].

A concern associated with CACXL is that it may be less effective than A-CXL or standard CXL due to the presence of an artificial lens, which acts as a barrier and potentially reduces and absorbs UV-A irradiance, thereby decreasing the efficacy of the procedure. However, Kormas et al. found that when both procedures were compared, they yielded similar results at a 12-month follow-up [17]. Another study comparing three methods of CXL (Standard epi-off CXL, CACXL, and transepithelial CXL) for progressive keratoconus across two years of follow-up, found that all three methods were similarly effective in halting the progression and achieving regression, with comparable visual and keratometric outcomes [25].

Other methods of CXL can be performed to increase corneal thickness through corneal stroma hydration or not removing the corneal epithelium, such as transepithelial CXL or hypo-osmolar CXL [10,13]. Hafezi et al. documented the progression of keratoconus after hypo-osmolar riboflavin CXL [10]. In their study, progression was halted and stabilized in 60% of eyes, and regression was achieved in 40% at a 6-month follow-up. Regarding transepithelial “epi-on” CXL, Spadea et al. demonstrated that a combination of riboflavin, along with substances such as ethylenediamine tetraacetic acid 0.01% and trometamol, can have a safe and positive impact on the degree of its penetration, while preserving the corneal epithelial layer and thickness [13]. This resulted in a significant decrease in the K apex (D) and gain in UDVA after 6-month follow-up. In contrast, no significant differences were observed between the 6-month and 12-month follow-ups for either group. On the other hand, no significant difference was observed in CDVA during the follow-up [13].

Knyazer et al. reported 1-year follow-up outcomes of A-CACXL treatment for keratoconus patients with thin corneas [26]. They reported on a significant improvement in various visual parameters following treatment, including UDVA, anterior steep keratometry, maximum keratometry, and anterior and posterior astigmatism, while no significant change was seen in MCT from baseline to the last follow-up visit [26]. In this study, we extended the follow-up time of the research conducted by Kormas et al., which evaluated a group of keratoconus patients with thin corneas after one year of follow-up post A-CACXL in comparison to a control group of patients with corneas thicker than 400 microns which received only A-CXL (5.4 J/cm^2^, 9 mW/cm^2^ for 10 min) [17]. Here, in terms of visual acuity, the control group showed a significant improvement in UDVA, while the A-CACXL group had a greater gain in CDVA. The magnitude of visual gain in our study is comparable to other studies with patients who underwent standard or accelerated CXL.

Keratometric measurements at the 3-year follow-up visit indicated stability in both groups. The K-mean front values remained stable across both groups, with no significant changes observed. These results suggest that both treatment modalities are effective in stabilizing keratometric parameters in the study groups.

The progression rates at 3 years were 13% (4 out of 32) for the control group and 27% (8 out of 30) for the case group, without a significant difference (*p* = 0.2, Figure 1, Table 4). There was a trend towards better stabilization in the A-CXL group compared to the A-CACXL group. This tendency may be attributed to the more advanced ectasia in the case group. Further studies with extended follow-up are needed to clarify this finding.

A wide range of studies have been published concerning the progression rates after CXL procedure in patients with progressive keratoconus, due to the diversity in baseline characteristics and follow-up periods [12,18,27,28,29]. Kuechler et al. reported that eyes with maximum keratometry values higher than 58.0 D had a 23% progression rate at one year follow-up [30]. On the other hand, the study introducing CACXL by Jacob et al. did not show any progression after the treatment [15]. Furthermore, nearly a third of the treated eyes had a regression [15]. A study by Hafezi et al. described a new modified CXL approach (“sub 400”) for treating thin corneas by adjusting the UVA fluence based on the individual corneal thickness [14]. The study included 39 eyes with corneal thickness between 214 and 398 µm. It showed a 90% success rate in halting the progression of keratoconus after a 12-month follow-up period, potentially expanding the range of corneal thickness values that can be treated with CXL. In addition, there was a significant reduction in K-max values.

In our study, we compared other parameters, such as keratometry, anterior astigmatism, ISV, IHD, and BAD-D, which can indicate treatment stabilization or progression in keratoconus across the study groups (Table 3). No significant changes were found. Other findings represent only a trend and are not statistically significant. These findings suggest that, overall, the treatment in the study groups was successful in stabilizing keratoectasia.

This study has several limitations. First, its retrospective design inherently relies on existing data and limits the ability to control for potential confounding variables. Second, the relatively small sample size in both groups may limit the reproducibility of our findings, highlighting the need for larger studies further to evaluate the non-inferiority of A-CACXL in thin corneas. Third, anterior segment optical coherence tomography was not routinely performed postoperatively, which limited the availability of structural outcome measures and the assessment of the corneal demarcation line after treatment. Moreover, patients who underwent A-CACXL presented with more advanced keratoconus at baseline, with higher Kmax values and thinner corneas, which may have influenced disease progression and outcomes. Finally, endothelial cell density (ECD) measurements were not performed consistently over the 3-year follow-up, precluding the ability to provide longitudinal ECD data.

According to current knowledge, our study is the first to examine and compare the clinical and tomographic characteristics of patients with keratoconus and MCT below 400 µm with those having MCT of 400 µm or greater following an A-CXL protocol over a 3-year follow-up. Our findings indicate that A-CACXL halted keratoconus progression in 73% of eyes and achieved regression in 37% of eyes, with rates comparable to those of A-CXL. These findings challenge the assumption that contact lenses can reduce a substantial amount of ultraviolet-A irradiance, may affect oxygen availability, and may impact the penetration and distribution of riboflavin in the cornea, thereby diminishing the effectiveness of CXL treatment. Furthermore, unlike the other methods described above, CA-CXL is simple and convenient to perform. It does not require any adjustments to existing methods, nor does it include calculations or special equipment. This reduces the risk of mistakes, which could affect the efficacy of the procedure and patient safety.

Both groups demonstrated visual improvement in UDVA and BCVA along with comparable keratometry changes during the three-year follow-up period. A-CACXL was performed in patients with more advanced keratoconus and demonstrated similar outcomes to A-CXL in those with less progressed keratoconus. Therefore, as indicated in our previous study, after a 1-year follow-up, our 3-year follow-up results provide strong evidence supporting A-CACXL as a safe and effective treatment option for patients with advanced keratoconus and MCT lower than 400 µm. Due to its procedural simplicity, lack of specialized equipment, and favorable safety profile, A-CACXL may represent a practical and effective treatment option for patients with thin corneas, especially in resource-limited settings. Considering this, additional research, particularly prospective, multicenter, randomized controlled trials are necessary to assess the non-inferiority and long-term stability of the A-CACXL protocol and to further elucidate the clinical and imaging outcomes associated with it.

## Figures and Tables

**Table 1:** Key features of the corneal cross-linking method employed in this study, aligned with the recommended standardized terminology and protocol nomenclature.

Parameter	Variable
**Treatment target**	Keratoconus
**Fluence (total) J/cm^2^**	5.4
**Soak time and interval (minutes)**	30 (q2)
**Intensity (mW)**	9
**Treatment time (minutes)**	10
**Epithelium status**	Off
**Chromophore**	Riboflavin 0.1% (Medio-Cross; Peschke Meditrade, Switzerland)
**Chromophore carrier**	Dextran 5% (Medio-Cross; Peshke Meditrade, Switzerland)
**Chromophore osmolarity**	Iso-osmolar
**Chromophore concentration**	0.1%
**Light source**	LightLink-CXL, LightMed, San Clemente, CA, USA
**Irradiation mode (interval)**	Continuous
**Protocol modifications**	Contact lens-assisted

**Table 2:** Characteristics of participants: Clinical and demographic information.

Characteristic	A-CACXL, N = 30	A-CXL, N = 32	* p * -Value
**Age** **Mean ± SD (N)**	25.2 ± 7.1 (30)	21.8 ± 4.5 (32)	**0.022**
**Sex, n (%)** **Female** **Male**	13 (43%) 17 (57%)	14 (44%) 18 (56%)	0.9
**Origin, n (%)** **Jewish** **Bedouin**	9 (30%) 21 (70%)	14 (44%) 18 (56%)	0.2
**Eye, n (%)** **Right** **Left**	15 (50%) 15 (50%)	11 (34%) 21 (66%)	0.2
**Kmax (D)** **Mean ± SD (N)**	61.0 ± 6.0	55.1 ± 4.2	**<0.001**
**Anterior Kmean (D)** **Mean ± SD (N)**	51.7 ± 4.3	47.7 ± 3.0	**<0.001**
**Anterior Astigmatism (D)** **Mean ± SD (N)**	5.06 ± 2.53	3.96 ± 1.94	**0.044**
**MCT (μm)** **Mean ± SD (N)**	398 ± 32	463 ± 31	**<0.001**
**BAD-D (D)** **Mean ± SD (N)**	12.4 ± 3.7	8.28 ± 2.96	**<0.001**
**Observational period (years)** **Mean ± SD (N)**	4.10 ± 1.42 (29)	3.72 ± 1.20 (30)	0.3

**Table 3:** A-CXL and A-CACXL preoperative, 1-year and 3-year follow-up values.

	A-CXL Group					A-CACXL Group				
	Baseline Mean ± SD n = 32	12 Months Mean ± SD n = 32	3 years Mean ± SD n = 32	12 Months *p*-Value	3 Years *p*-Value	Baseline Mean ± SD n=30	12 Months Mean ± SD n = 30	3 Years Mean ± SD n = 30	12 Months *p*-Value	3 Years *p*-Value
**UDVA (LogMAR)**	0.63 ± 0.41	0.43 ± 0.31	0.44 ± 0.28	**0.029**	**0.031**	0.93 ± 0.58	0.74 ± 0.49	0.66 ± 0.51	0.2	0.056
**BCVA (LogMAR)**	0.32 ± 0.18	0.24 ± 0.15	0.18 ± 0.15	0.09	**0.001**	0.51 ± 0.30	0.40 ± 0.49	0.33 ± 0.34	0.3	**0.037**
**Kmax (D)**	55.1 ± 4.2	55.0 ± 4.2	54.5 ± 3.9	0.9	0.5	61.0 ± 6.0	60.2 ± 6.6	60.2 ± 6.6	0.7	0.8
**K_mean_front (D)**	47.65 ± 2.97	47.88 ± 2.95	47.46 ± 2.86	0.7	0.8	51.7 ± 4.3	51.9 ± 4.4	51.8 ± 4.5	0.8	0.9
**K steep front (D)**	49.74 ± 3.37	49.72 ± 3.38	49.26 ± 3.24	0.9	0.6	54.5 ± 4.6	54.4 ± 5.0	54.1 ± 4.9	0.9	0.7
**K_flat_front (D)**	45.78 ± 2.89	46.22 ± 2.83	45.81 ± 2.82	0.5	0.9	49.4 ± 4.1	49.8 ± 4.1	49.7 ± 4.3	0.7	0.8
**Anterior Astigmatism (D)**	3.96 ± 1.94	3.52 ± 1.96	3.47 ± 1.86	0.4	0.3	5.06 ± 2.53	4.66 ± 2.46	4.20 ± 2.41	0.5	0.2
**K steep back (D)**	−7.40 ± 0.65	−7.48 ± 0.68	−7.49 ± 0.73	0.6	0.6	−8.19 ± 0.94	−8.16 ± 0.96	−8.23 ± 1.01	0.9	0.9
**K_flat_back (D)**	−6.75 ± 0.59	−6.84 ± 0.54	−6.71 ± 0.61	0.5	0.8	−7.26 ± 0.79	−7.35 ± 0.83	−7.38 ± 0.92	0.7	0.6
**MCT (μm)**	463 ± 31	450 ± 35	453 ± 31	0.1	0.2	398 ± 32	388 ± 41	381 ± 44	0.3	0.07
**ISV (μm)**	90 ± 29	87 ± 29	84 ± 28	0.7	0.4	108 ± 31	104 ± 32	102 ± 34	0.6	0.5
**IHD (μm)**	0.11 ± 0.05	0.13 ± 0.05	0.15 ± 0.13	0.1	0.1	0.12 ± 0.06	0.14 ± 0.07	0.13 ± 0.08	0.4	0.6
**BAD-D**	8.28 ± 2.96	8.52 ± 2.91	8.40 ± 2.43	0.7	0.9	12.4 ± 3.7	12.4 ± 3.8	13.0 ± 4.0	0.9	0.5

**Table 4:** Progression and regression of 3-year follow-up in study populations.

	A-CACXL, N = 30	A-CXL, N = 32	*p*-Value
**Progression, n (%)**	8 (27%)	4 (13%)	0.2
**Regression, n (%)**	11 (37%)	11 (34%)	>0.9

## Data Availability

The data presented in this study are available on request from the corresponding author. The data are not publicly available due to privacy issues.

## Abbreviations

The following abbreviations are used in this manuscript:

CXL	Corneal Cross-Linking
A-CACXL	Contact lens-assisted accelerated corneal cross-linking
Kmax	Maximum keratometry
D	Diopters
Kmean	Mean keratometry
K	Keratometry
MCT	Minimum corneal thickness
BAD-D	Belin–Ambrósio enhanced ectasia display total deviation
UDVA	Uncorrected distance visual acuity
BCVA	Best-corrected visual acuity
ISV	Index of surface variance
IHD	Index of height decentration
